# *WWOX* Mutation as a Rare Cause of Neonatal‐Infantile Parkinsonism Mimicking a Neurotransmitter Disorder: A Case Report

**DOI:** 10.1111/jpc.70401

**Published:** 2026-05-06

**Authors:** Ozge Serce Pehlevan, Gizem Gider Yaman, Anıl Gok, Leman Tekin Orgun

**Affiliations:** ^1^ Faculty of Medicine, Department of Neonatology, Division of Pediatrics Kocaeli University Kocaeli Türkiye; ^2^ Faculty of Medicine, Department of Pediatric Neurology, Division of Pediatrics Kocaeli University Kocaeli Türkiye

**Keywords:** dopaminergic dysfunction, infantile parkinsonism, neonatal parkinsonism, neurotransmitter disorder, *WWOX*

## Abstract

- Neonatal‐onset parkinsonism is extremely rare and is most often attributed to monoamine neurotransmitter synthesis or metabolism disorders rather than to primary neurodevelopmental diseases.
- *WWOX* mutations should be considered in the differential diagnosis of neonatal hypokinetic–rigid syndromes, particularly when metabolic and neurotransmitter studies are normal.
- *WWOX*‐related parkinsonian features likely reflect a developmental circuitopathy, characterized by impaired maturation and organization of dopaminergic and inhibitory networks rather than dopaminergic neuronal degeneration.
- Clinical differentiation between neurotransmitter disorders and *WWOX*‐related parkinsonism is challenging, as both may present with neonatal rigidity, hypokinesia, hypotonia, abnormal movements, and seizures.
- Early comprehensive genetic testing is essential in neonates with parkinsonian features when standard metabolic investigations are unrevealing, as it may reveal rare but clinically relevant neurodevelopmental etiologies.

## 1 Introduction

Neonatal‐onset parkinsonism is an exceptionally rare and diagnostically challenging condition in which hypokinetic–rigid features most commonly result from defects in neurotransmitter biosynthesis or metabolism rather than the primary dopaminergic neurodegeneration observed in adults. Impairments in dopamine and serotonin pathways lead to monoamine deficiency, clinically manifesting as dystonia, oculogyric crises, and bradykinesia [1]. This condition is broadly classified into developmental and degenerative forms. The former results from impaired maturation of dopaminergic circuitry, whereas the latter is characterized by progressive nigrostriatal degeneration and may occur as part of complex multisystem neurodevelopmental disorders [2].

WW domain‐containing oxidoreductase (*WWOX*) is a key regulator of neural development, synaptic homeostasis, and cortical organization. However, its role in monoamine pathways remains not well defined. Experimental studies suggest that *WWOX* modulates the vulnerability of dopaminergic neurons through interactions with JNK1 (stress‐related kinase), particularly, which is involved in neurodegenerative signaling cascades [2]. Furthermore, genome‐wide association studies have identified variants at the *WWOX* locus as genetic modifiers associated with accelerated progression to dementia in Parkinson's disease, suggesting a modulatory role in dopaminergic resilience rather than primary neurodegeneration [3].

Pathogenic *WWOX* variants are well‐established causes of severe neurodevelopmental disorders including SCAR12 and WOREE (*WWOX*‐related epileptic encephalopathy). However, a parkinsonian phenotype has not been reported in humans to date. Here, we describe the first case of neonatal–infantile parkinsonism associated with a pathogenic *WWOX* variant, clinically mimicking a primary monoamine neurotransmitter synthesis defect.

## 2 Case Presentation

A male neonate was born at 38 weeks of gestation via spontaneous vaginal delivery to a 28‐year‐old gravida 6, para 4 woman with a history of one spontaneous abortion and one elective termination. There were no known maternal comorbidities during pregnancy. The delivery was meconium‐stained and required approximately 15 s of positive pressure ventilation. APGAR scores were 6 and 8 at 1 and 5 min, respectively. The birth weight was 3820 g, and the head circumference was 35 cm (50th–95th percentile). Although parental consanguinity was not present, both parents were from the same village. There was no history of epilepsy, neurodevelopmental disorders or other neurological conditions.

**FIGURE 1:**
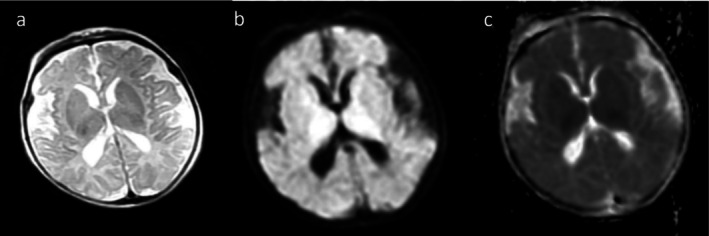
Brain MRI; (a) Axial diffusion‐weighted imaging demonstrates a small hyperintense diffusion‐restricted focus in the left lateral thalamic region. (b) The corresponding Apparent Diffusion Coefficient (ADC) map shows a hypointense signal consistent with true diffusion restriction. (c) Axial FLAIR sequence reveals bilateral frontotemporal atrophy, corpus callosum hypoplasia, and mild enlargement of the lateral ventricles.

The neonate was admitted to the neonatology unit at an external facility due to respiratory distress and abnormal neurological findings on examination (axial hypotonia with peripheral hypertonia, alternating abnormal posturing, and reduced spontaneous limb movements). He has slightly dysmorphic features, including a narrow forehead, thin lips, full cheeks, almond‐shaped eyes, a slightly elevated palate, a long philtrum, an umbilical hernia, and a sacral Mongolian spot. A right clavicle fracture was noted. Echocardiography revealed a secundum atrial septal defect and a small patent ductus arteriosus. Cranial and abdominal ultrasound examinations were unremarkable. Diffusion‐weighted brain MRI showed a small diffusion‐restricted area in the left lateral thalamus with corresponding low apparent diffusion coefficient and mild ventricular dilatation (Figure 1a,b). These findings were attributed to his birth history. The patient was discharged home against medical advice on postnatal day 12.

One day later, he was brought to our emergency department and subsequently admitted to the NICU because of poor feeding and focal twitching involving the perioral region. Neurological examination revealed axial hypotonia, hypokinesia of the extremities, facial hypomimia, a hypoactive Moro reflex, and cortical fisting of the thumbs. During hospitalization, he exhibited paroxysmal, alternating tone abnormalities lasting from minutes to hours, characterized by fluctuating hypotonia and hypertonia and prolonged periods of hypokinesia. He also experienced episodes of spinal rigidity triggered by tactile or auditory stimuli.

**FIGURE 2:**
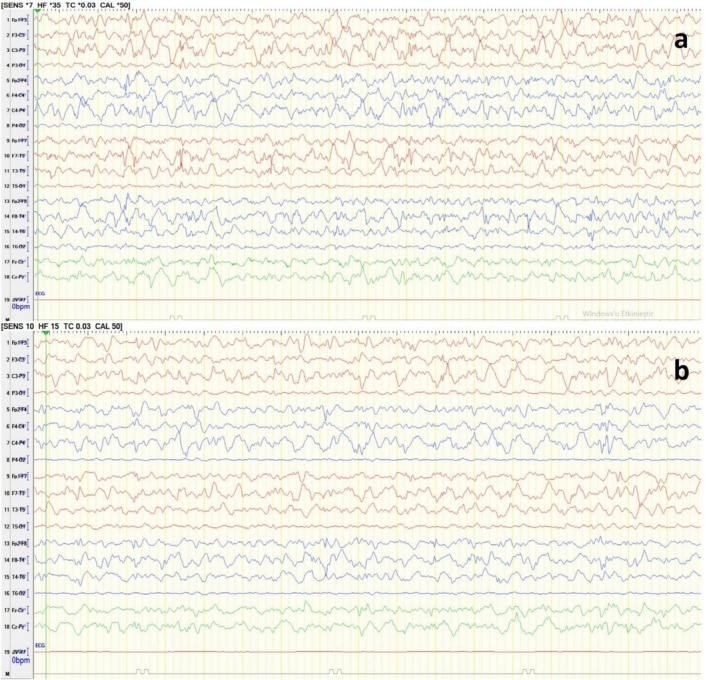
EEG; (a) EEG during recurrent epileptic spasms before treatment optimization. The interictal EEG demonstrates multifocal sharp and slow waves, along with disruption of the background rhythm. (b) Control EEG exhibits a significant resolution of epileptiform discharges, but with impaired background organization.

On day 14, stereotyped movements, such as tongue thrusting and bicycling of the lower limbs, were observed. Video‐EEG demonstrated isolated, infrequent, low‐amplitude, multifocal sharp waves, predominantly in the right hemisphere (Figure 2a). Phenobarbital was initiated with a loading dose followed by maintenance therapy at 5 mg/kg/day, resulting in a marked reduction in seizure‐like events. Auditory brainstem response testing and thyroid function tests were within normal limits. Brain MRI demonstrated nonspecific structural abnormalities, including bilateral frontotemporal atrophy, corpus callosum hypoplasia, and enlargement of the lateral ventricles (Figure 1c). Ophthalmologic evaluation showed no signs of optic atrophy or additional structural abnormalities. A comprehensive metabolic evaluation, including serum ammonia and creatine phosphokinase levels, blood gas analysis, acylcarnitine profile, plasma and urine amino acids, and urine organic acids, revealed no abnormalities. Given the persistence of hypotonia, abnormal movement patterns including alternating hypokinetic–rigid episodes, and seizures, a neurotransmitter disorder was considered a preliminary diagnosis. Whole‐exome sequencing identified a homozygous WWOX variant, NM_016373.4:c.716T>G, resulting in the predicted amino acid substitution p.(Leu239Arg). Cerebrospinal fluid (CSF) neurotransmitter analysis was not performed. He was discharged in stable condition on postnatal day 23, with a scheduled follow‐up in paediatric neurology.

Following routine immunization 1 month after discharge, the patient developed recurrent flexor spasms, occurring 20–30 times per day. EEG demonstrated multifocal sharp waves. Initially, vigabatrin was added to phenobarbital; however, the patient continued to experience epileptic spasms despite this combination. After valproic acid was introduced, the frequency of spasms decreased, but the spasms did not fully resolve. Following the addition of clobazam, the spasms completely subsided, and the EEG showed marked improvement with a significant reduction in epileptiform discharges, although the background activity remained disorganized. (Figure 2b) The patient has since remained seizure‐free on a regimen of valproic acid and clobazam. At the most recent evaluation (age 4 months), the patient had been seizure‐free for 1 month on valproic acid and clobazam; however, his hypokinetic movement disorder and parkinsonian symptoms persisted.

## 3 Discussion

Although early‐onset seizures and movement abnormalities are well described in *WWOX*‐related disorders, the hypokinetic–rigid features observed in our patient during follow‐up have not previously been recognized as part of the *WWOX* phenotype. Our findings are consistent with developmental, nondegenerative parkinsonism, characterized by impaired maturation of dopaminergic networks and synaptic dysfunction rather than neuronal loss.

*WWOX* is not traditionally considered a gene involved in dopamine synthesis or transport. However, evidence from knockout models and human brain organoids indicates that *WWOX* plays a critical role in neuronal maturation, synaptic regulation, and the integrity of dopaminergic circuitry [4, 5]. *WWOX* deficiency may clinically resemble infantile parkinsonism–dystonia, presenting with early‐onset rigidity and bradykinesia. However, the underlying mechanism reflects abnormal neuronal maturation and circuit organization rather than dopaminergic degeneration [4]. In models of acquired parkinsonism such as those induced by MPP^+^, *WWOX* expression undergoes dynamic changes during dopaminergic neurodegeneration, suggesting that, beyond its developmental role, *WWOX* may also exert neuroprotective or modulatory effects in degenerative contexts [3, 4, 6]. Consistent with this, rodent *WWOX* knockout models demonstrate profound neurodevelopmental abnormalities including neuronal migration defects, hypomyelination, interneuron loss, cortical disorganization, and seizures [4, 5].

These models also exhibit GSK‐3β hyperactivation, a kinase involved in tau regulation and synaptic stability. Conversely, GSK‐3β inhibition (e.g., with lithium) has been shown to reduce seizure susceptibility [7]. Dysregulated GSK‐3β signaling further affects dopaminergic transmission, receptor trafficking, and synaptic plasticity [7, 8, 9]. *WWOX* mutations are associated with a broad phenotypic spectrum in humans, ranging from the milder ataxic form (SCAR12) to severe epileptic encephalopathy (WOREE syndrome) [4]. Human *WWOX*‐knockout brain organoids demonstrate hyperexcitability, gliosis, impaired DNA repair, and cortical disorganization, supporting a developmental rather than metabolic disease mechanism [4].

Environmental factors may further modulate disease expression. Neonatal stress has been shown to impair glymphatic system maturation through disruption of AQP4 polarization, leading to α‐synuclein accumulation and increased dopaminergic vulnerability [10]. In our patient, perinatal factors such as meconium‐stained amniotic fluid and clavicular fracture indicating perinatal stress may have exacerbated glial dysfunction and dopaminergic imbalance in the context of *WWOX* deficiency [10].

Clinically, distinguishing *WWOX*‐related parkinsonism from classical neurotransmitter disorders is challenging, as both may present with neonatal rigidity, hypokinesia, hypotonia, and feeding difficulties. However, metabolic investigations are typically normal in *WWOX* deficiency, highlighting the importance of early genetic testing for accurate diagnosis [4]. Genetic testing plays a critical role in the management of epileptic encephalopathies by enabling early etiological diagnosis, avoiding unnecessary investigations, and guiding prognosis and counseling.

In *WWOX*‐related disorders, early genetic confirmation may influence treatment strategies, facilitate anticipation of disease progression, and prevent diagnostic delays, particularly in cases mimicking neurotransmitter disorders. Moreover, it may reduce the need for invasive procedures such as lumbar puncture required for CSF neurotransmitter analysis. Notably, CSF neurotransmitter testing requires strict pre‐analytical conditions including rapid processing, immediate freezing, and transport under controlled conditions to specialized laboratories, which may limit its feasibility in routine clinical practice. Therefore, genetic testing represents a more accessible and less invasive diagnostic alternative, enabling a more targeted clinical approach. In addition, it supports precision medicine strategies and informs recurrence risk for families.

In our case, CSF neurotransmitter analysis could not be performed due to lack of availability in our center. Therefore, genetic testing was prioritized. Whole‐exome sequencing identified a homozygous *WWOX* variant (NM_016373.4:c.716T>G; p.Leu239Arg). Segregation analysis confirmed that both parents were heterozygous carriers of the variant. This variant was classified as likely pathogenic according to the American College of Medical Genetics and Genomics (ACMG) criteria, based on PM2 (absence in population databases), PM3 (detected in trans in carrier parents), and PP3_Moderate (multiple computational tools supporting a deleterious effect on protein function at a moderate level of evidence), with additional support from phenotypic similarity to previously reported *WWOX* cases [11, 12]. A two‐star pathogenic ClinVar entry further supports this classification [13]. However, PM5 was not applied in accordance with current recommendations.

The same *WWOX* variant has previously been reported in association with acquired microcephaly and severe epileptic encephalopathy without prominent parkinsonian features [14]. The hypokinetic–rigid manifestations observed in our patient suggest a potential expansion of the phenotypic spectrum associated with this variant.

To date, *WWOX* mutations have not been reported to present with a phenotype mimicking a neurotransmitter disorder. To our knowledge, this is the first reported case of neonatal parkinsonism presenting with features resembling a monoamine synthesis defect in which a pathogenic *WWOX* variant was identified. These findings expand the current understanding of early‐life parkinsonism to include neurodevelopmental circuitopathies with downstream effects on neurotransmitter systems.

## 4 Conclusion

This case highlights a *WWOX* mutation as a rare and nonclassical cause of neonatal–infantile parkinsonism, expanding the current understanding of early‐onset hypokinetic–rigid syndromes beyond classical monoamine neurotransmitter disorders. While congenital neurotransmitter defects typically result from enzymatic disruption of dopamine or serotonin synthesis, *WWOX* deficiency represents a developmental circuitopathy in which impaired neuronal maturation, GSK‐3β dysregulation, and glial dysfunction collectively disrupt dopaminergic function [4]. Perinatal stressors may have further contributed to increased dopaminergic vulnerability in this case [10].

In the absence of a relevant family history and with a normal metabolic profile, this case underscores the diagnostic and clinical importance of early comprehensive genetic testing in neonates presenting with rigidity, hypokinesia, or parkinsonian features. Recognition of *WWOX*‐associated developmental parkinsonism bridges the gap between neurotransmitter disorders and structural neurodevelopmental conditions, providing new insight into the neurobiological spectrum of early‐life parkinsonism.

## Funding

The authors have nothing to report.

## Ethics Statement

According to the policies of the ethics committee of Kocaeli University, single‐patient case reports that do not contain identifiable personal information do not require formal ethics committee approval. Therefore, ethical approval was not sought for this report. All procedures related to this case were carried out in accordance with institutional guidelines and ethical standards consistent with the Declaration of Helsinki.

## Consent

Written informed consent for publication was obtained from the patient's parents.

## Conflicts of Interest

The authors declare no conflicts of interest.

## Data Availability

Data sharing not applicable to this article as no datasets were generated or analysed during the current study.
